# Serum phosphate level at initiation of dialysis is associated with all-cause mortality: a multicenter prospective cohort study

**DOI:** 10.1080/0886022X.2018.1499530

**Published:** 2018-08-28

**Authors:** Akiko Owaki, Daijo Inaguma, Isao Aoyama, Shinichiro Inaba, Shigehisa Koide, Eri Ito, Kazuo Takahashi, Hiroki Hayashi, Midori Hasegawa, Yukio Yuzawa, AICOPP group

**Affiliations:** a Tosei General Hospital, Seto, Aichi, Japan;; b The Aichi Cohort Study of Prognosis in Patients Newly Initiated into Dialysis (AICOPP) Group;; c Fujita Health University School of Medicine, Toyoake, Aichi, Japan;; d Japan Community Health Care Organization Chukyo Hospital, Nagoya, Aichi, Japan

**Keywords:** Phosphate, dialysis initiation, all-cause mortality, multicenter cohort study, phosphate binder

## Abstract

**Introduction:** As glomerular filtration rate (GFR) decreases, serum phosphate level increases. Previous reports indicated that serum phosphate level was associated with mortality in patients on dialysis. However, few reports have examined the association using dialysis initiation as the baseline period.

**Methods:** This was a multicenter prospective cohort analysis including 1492 patients. Patients were classified into four quartiles based on the serum phosphate level at dialysis initiation, with Q1 being the lowest and Q4 the highest. All-cause mortality after dialysis initiation was compared using the log-rank test. The propensity score represented the probability of being assigned to group Q1 or Q2–4. All-cause mortality was compared in propensity score-matched patients by using the log-rank test for Kaplan–Meier curves. All-cause mortality of Q1 was compared with that for Q2–4 using multivariate Cox proportional hazard regression analysis. All-cause mortality was also determined among stratified groups with or without use of phosphate binders.

**Results:** Significant differences in cumulative survival rates were observed between the four groups (*p* < .001). After propensity score-matching, mortality was significantly higher in the Q1 group than the Q2-4 group (*p* = .046). All-cause mortality was significantly higher in the Q1 group after adjustment for history of CAD (hazard ratio [HR] = 0.76, 95% confidence interval [CI]: 0.58 − 1.00, *p* = .048). However, there was no significant difference between the two groups after adjustment for estimated GFR.

**Conclusion:** The serum phosphate level at the time of dialysis initiation was associated with all-cause mortality. However, the serum phosphate level was dependent on the renal function.

## 1. Introduction

The concept of chronic kidney disease-mineral and bone disorders (CKD-MBD) focuses on survival prognosis rather than the bone lesions, and management of serum phosphate and calcium levels is considered to be especially important [1]. The cause of central pathogenesis in CKD-MBD is vascular calcification. An increase in serum phosphate level leads to vascular calcification, which is associated with cardiovascular events and mortality in patients on maintenance dialysis [2–4]. Previous reports showed that a decrease in serum phosphate level was also associated with higher mortality because hypophosphatemia represented a poor nutritional state [5–7]. Accordingly, target guidelines were set for the serum phosphate levels. The guidelines recommend that serum phosphate level should be maintained within the normal range in pre-dialysis patients. However, many guidelines based on CKD-MBD set 3.5–6.0 mg/dL as the target range for serum phosphate level [8–11].

As the glomerular filtration rate (GFR) decreases, serum phosphate level increases, and becomes especially significant in CKD stage G4 [12]. Moreover, it is often difficult to manage serum phosphate levels at the time of dialysis initiation. However, few reports have examined the association between serum phosphate level at dialysis initiation and mortality during the maintenance dialysis. The purpose of this study was to clarify this association.

## 2. Materials and methods

### 2.1. Subjects

The participants were selected from among the patients who started the dialysis between October 2011 and September 2013 at 17 institutions affiliated with the AICOPP group. The inclusion criteria were CKD, an age of ≥20 years, and written consent. After excluding patients who discontinued dialysis or died while in the hospital, in addition to those who refused to be enrolled, 1,520 patients remained. We excluded 28 patients whose serum phosphate levels at dialysis initiation were not measured. Thus, we included 1,492 patients in the final analysis. We determined survival prognosis as of 30 September 2016, by conducting a survey.

### 2.2. Patient characteristics and data at dialysis initiation (baseline)

Using the first dialysis session as the baseline time point, we compared the values of variables. Body mass index (BMI) was measured at the first hemodialysis session or the first exchange of peritoneal dialysate. Diabetes was defined as fasting blood glucose ≥126 mg/dL, random blood glucose ≥200 mg/dL, HbA1c (National Glycohemoglobin Standardization Program) ≥ 6.5%, use of insulin, or use of oral hypoglycemic agents. History of coronary artery disease (CAD) was defined as having a history of coronary artery intervention, heart bypass surgery, or ischemic changes on electrocardiogram. History of ischemic stroke was defined as confirmed diagnoses using computed tomography or magnetic resonance imaging. Medication use referred to the drugs being taken at the time of dialysis initiation. Blood tests were performed on samples taken before the first hemodialysis session or the first exchange of peritoneal dialysate. Blood pressure was measured before the first dialysis session or the first exchange of peritoneal dialysate.

### 2.3. Classification of patients by quartiles

Based on serum phosphate level, patients were classified into four quartiles (Q1, Q2, Q3, and Q4). There were 360–388 patients in each group. We compared the association between measured parameters and all-cause mortality. We further classified patients into two groups (Q1 and Q2–4) using the propensity score-matching.

### 2.4. Survey of survival prognosis

Outcomes were determined by reviewing the medical records of the AICOPP group institutions or by corresponding with the facilities where patients had been transferred for maintenance dialysis. The correspondence included a questionnaire for assessing the outcomes.

### 2.5. Outcomes

The study outcomes were all-cause mortality rates in the four groups based on quartiles of serum phosphate level, and all-cause and cardiovascular-related mortality rates in the two propensity score-matched groups. Cardiovascular-related death was defined as deaths due to heart failure, acute coronary syndrome, fatal arrhythmia, stroke, or peripheral artery disease.

### 2.6. Statistical processing

SPSS statistics version 24 and the Easy R program [13] were used for statistical processing. Patient characteristics and baseline data were compared between the four groups and the propensity score-matched groups using analysis of variance for continuous variables and Fisher’s exact test for nominal variables. The propensity score, which we calculated using logistic regression models, represented the probability that a patient would be assigned to the Q1 group or the Q2–4 group and additionally the Q4 group or the Q1–3 group (PS Q4 cohort). Using a propensity-score-matching procedure, the two groups were similarly distributed, indicating that the differences in covariates between the groups were minimized. The incidence of mortality was compared using the log-rank test for Kaplan–Meier curves. The factors that contributed to all-cause mortality were examined using univariate regression analysis. We conducted multivariate Cox proportional hazards analysis using models 1–4 with adjustment for age, gender, history of CAD, and estimated GFR (eGFR). Continuous variables were expressed as the mean and standard deviation, or the median and interquartile range, and categorical variables were presented as a percentage. The *p* values of less than 5% were considered statistically significant.

## 3. Results

### 3.1. Comparison of patient characteristics and baseline data

**Figure 1.**
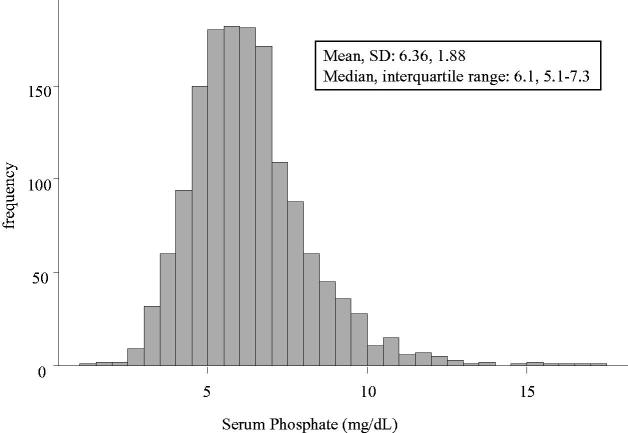
Distribution of serum phosphate at dialysis initiation.

Table 1 shows patient characteristics and baseline data for the four groups. Significant differences were observed between the groups in age, BMI, diastolic blood pressure, laboratory data (eGFR; hemoglobin, serum albumin, blood urea nitrogen, serum creatinine, serum adjusted calcium, serum phosphate, and serum magnesium levels), use of calcium carbonate, and use of an erythropoiesis-stimulating agent. Serum phosphate level showed a normal distribution: mean 6.36, standard deviation 1.88, median 6.1, and interquartile range 5.1–7.3 (Figure 1).

**Table 1.** Baseline characteristics and laboratory data.

Variables	Alln, 1,492	Q1n, 388	Q2n, 368	Q3n, 360	Q4n, 376	*p* value
Age (years old)	67.5, 13.0	72.3, 10.7	68.0, 12.5	66.9, 12.7	62.7, 14.2	< .01
Female gender	484, 32.4	121, 31.2	121, 32.9	113, 31.4	129, 34.3	.779
BMI (kg/m^2^)	23.5, 4.4	23.1, 3.9	23.5, 4.4	23.2, 4.1	24.2, 5.0	.03
SBP (mmHg)	151, 26	149, 28	154, 25	151, 26	151, 26	.51
DBP (mmHg)	77, 15	74, 15	77, 14	78, 14	79, 16	< .01
Diabetes Mellitus	762, 51.1	204, 52.6	193, 52.4	191, 53.1	174, 46.3	.199
History of CAD	247, 16.6	99, 25.5	56, 15.2	54, 15.0	38, 10.1	< .01
History of ischemic stroke	203, 13.6	72, 18.6	52, 14.1	47, 13.1	32, 8.5	< .01
Laboratory data						
Hemoglobin (g/dL)	9.4, 1.5	9.5, 1.4	9.5, 1.5	9.4, 1.5	9.0, 1.7	< .01
Albumin (g/dL)	3.20, 0.60	3.20, 0.59	3.27, 0.60	3.23, 0.58	3.12, 0.61	.10
Alkaline phosphatase (IU/L)	264, 174	276, 132	253, 120	254, 148	272, 258	.163
Uric acid (mg/dL)	8.8, 2.4	8.3, 2.2	8.7, 2.2	8.7, 2.4	9.5, 2.7	< .01
BUN (mg/dL)	91.8, 30.4	77.2, 23.7	86.9, 25.9	93.8, 24.6	109.9, 35.7	< .01
Creatinine (mg/dL)	8.97, 3.16	7.30, 2.04	8.45, 2.16	9.11, 2.67	11.08, 4.04	< .01
eGFR (ml/min/1.73m^2^)	5.4, 2.2	6.6, 2.9	5.5, 1.9	5.1, 1.4	4.4, 1.6	< .01
Adjusted calcium (mg/dL)	8.6, 1.1	9.0, 0.8	8.7, 0.9	8.5, 1.0	8.3, 1.3	< .01
Phosphorus (mg/dL)	6.4, 1.9	4.4, 0.6	5.7, 0.3	6.7, 0.3	8.8, 1.6	< .01
Magnesium (mg/dL)	2.1, 0.5	2.1, 0.4	2.1, 0.4	2.1, 0.5	2.2, 0.5	< .01
LDL-cholesterol (mg/dL)	90, 34	83, 31	91, 36	91, 35	94, 35	< .01
HDL-cholesterol (mg/dL)	45, 17	44, 16	46, 17	44, 15	45, 18	.241
Triglyceride (mg/dL)	125, 71	116, 56	124, 67	127, 82	133, 75	.23
CRP (mg/dL)	1.8, 4.1	1.7, 3.8	1.8, 4.4	1.6, 4.3	2.1, 3.9	.335
Ferritin (ng/mL)	222, 995	228, 654	175, 261	176, 278	308, 1832	< .01
Intact PTH (pg/mL)	354, 292	284, 196	330, 239	367, 272	431, 398	< .01
1,25-dihydroxyvatamin D (pg/mL)	13.6, 7.6	15.6, 7.1	14.0, 8.3	13.2, 7.4	11.7, 7.3	< .01
Bicarbonate (mmol/L)	19.6, 4.9	21.0, 4.4	20.2, 4.4	19.1, 4.8	18.0, 5.5	< .01
Medication						
ACEIs or ARBs	898, 60.2	238, 61.3	240, 65.2	209, 58.1	211, 56.1	.56
Calcium channel blockers	1181, 79.2	299, 77.1	302, 82.1	293, 81.4	287, 76.3	.120
β blockers	519, 34.8	150, 38.7	119, 32.3	135, 37.5	115, 30.6	.54
Loop diuretics	980, 65.7	255, 65.7	229, 62.2	253, 70.3	243, 64.6	.138
Statins	600, 40.2	171, 44.1	149, 40.5	146, 40.6	134, 35.6	.127
Calcium carbonate	524, 35.1	109, 28.1	145, 39.4	137, 38.1	133, 35.4	.05
Vitamin D receptor activators	408, 27.3	116, 29.9	100, 27.2	101, 28.1	91, 24.2	.356
Sodium bicarbonate	665, 44.6	179, 46.1	167, 45.4	155, 43.1	164, 43.6	.812
ESAs	1289, 86.4	352, 90.7	321, 87.2	313, 86.9	303, 80.6	.02

Mean, SD Value, %.

BMI: body mass index; SBP: systolic blood pressure; DBP: diastolic blood pressure; CAD: coronary artery disease; CVD: cardiovascular disease; BUN: blood urea nitrogen; eGFR: estimated glomerular filtration rate; LDL: low density lipoprotein; HDL: high density lipoprotein; CRP: C reactive protein; PTH: parathyroid hormone; ACEI: angiotensin converting enzyme inhibitor; ARB: angiotensin 1 receptor blocker; ESA: erythropoiesis stimulating agent.

### 3.2. Comparison of all-cause mortality between the four groups

**Figure 2.**
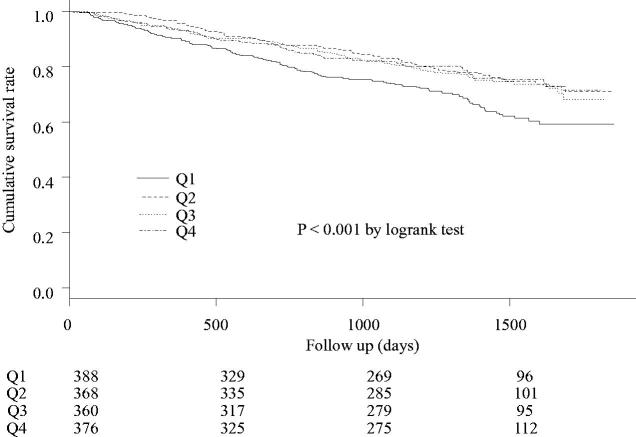
All-cause mortality in the four groups significant differences were observed between the four groups’ cumulative survival rates (*p* < .001).

Figure 2 compares all-cause mortality between the four groups. The median and interquartile range of the follow-up period was 1,282 (982–1,522) days. There were 503 all-cause deaths during the follow-up period [Q1 group, 130 deaths (33.5%); Q2 group, 83 deaths (22.6%); Q3 group, 87 deaths (24.2%); Q4 group, 84 deaths (22.3%)]. Significant differences were observed in the cumulative survival rates between the four groups (*p* < .001).

### 3.3. Comparison of patient characteristics and baseline data among the propensity score-matched cohorts

There were 368 patients in each group. Table 2 shows the patient characteristics and baseline data in the two groups after propensity score-matching. There were no significant differences in variables except for the history of CAD, the renal function including eGFR, cardiothoracic ratio, serum intact parathyroid hormone, serum 1,25-dihydroxyvitamin D, and bicarbonate level. Supplementary Table shows the patient characteristics and baseline data in the PS Q4 cohort.

**Table 2.** Baseline characteristics and laboratory data after PS matching.

Variables	Q1n, 368	Q2-Q4n, 368	*p* value
Age (years old)	72.0, 10.4	72.0, 10.4	1.000
Female gender	110, 29.9	110, 29.9	1.000
BMI (kg/m^2^)	23.2, 3.9	23.2, 3.7	.863
SBP (mmHg)	149, 28	152, 25	.101
DBP (mmHg)	74, 15	75, 14	.322
Diabetes Mellitus	196, 53.3	188, 51.1	.606
History of CAD	93, 25.3	63, 17.1	.007
History of ischemic stroke	70, 19.0	55, 14.9	.169
Laboratory data			
Hemoglobin (g/dL)	9.5, 1.4	9.3, 1.4	.072
Albumin (g/dL)	3.21, 0.57	3.21, 0.58	.963
Alkaline phosphatase (IU/L)	278, 134	260, 145	.089
Uric acid (mg/dL)	8.3, 2.2	8.7, 2.4	.016
BUN (mg/dL)	77.0, 23.7	91.8, 26.9	< .001
Creatinine (mg/dL)	7.29, 2.05	8.50, 2.31	< .001
eGFR (ml/min/1.73m^2^)	6.7, 2.9	5.5, 1.9	< .001
Adjusted calcium (mg/dL)	9.0, 0.8	8.6, 0.9	< .001
Phosphorus (mg/dL)	4.3, 0.7	6.0, 1.0	< .001
Magnesium (mg/dL)	2.1, 0.4	2.1, 0.5	.378
LDL-cholesterol (mg/dL)	83, 31	88, 35	.054
HDL-cholesterol (mg/dL)	44, 16	45, 16	.531
Triglyceride (mg/dL)	116, 56	128, 94	.050
CRP (mg/dL)	1.7, 3.9	2.2, 5.4	.128
Ferritin (ng/mL)	228, 672	281, 1862	.622
Intact PTH (pg/mL)	287, 200	341, 231	.002
1,25-dihydroxyvatamin D (pg/mL)	15.8, 7.2	13.9, 8.1	.046
Bicarbonate (mmol/L)	21.0, 4.5	19.8, 4.8	.001
Medication			
ACEIs or ARBs	226, 61.4	231, 62.8	.648
Calcium channel blockers	285, 77.4	301, 81.8	.170
β blockers	144, 39.1	123, 33.4	.125
Loop diuretics	240, 65.2	240, 65.2	1.000
Statins	163, 44.3	147, 39.9	.263
Calcium carbonate	103, 28.0	122, 33.2	.150
Vitamin D receptor activators	112, 30.4	96, 26.1	.219
Sodium bicarbonate	172, 46.7	168, 45.7	.824
ESAs	334, 90.8	323, 87.8	.234

Mean, SD Value, %.

BMI: body mass index; SBP: systolic blood pressure; DBP: diastolic blood pressure; CAD: coronary artery disease; CVD: cardiovascular disease; BUN: blood urea nitrogen; eGFR: estimated glomerular filtration rate; LDL: low density lipoprotein; HDL: high density lipoprotein; CRP: C reactive protein; PTH: parathyroid hormone; ACEI: angiotensin converting enzyme inhibitor; ARB: angiotensin 1 receptor blocker; ESA: erythropoiesis stimulating agent.

### 3.4. Comparison of all-cause and cardiovascular-related mortality in the two groups after propensity score-matching

**Figure 3.**
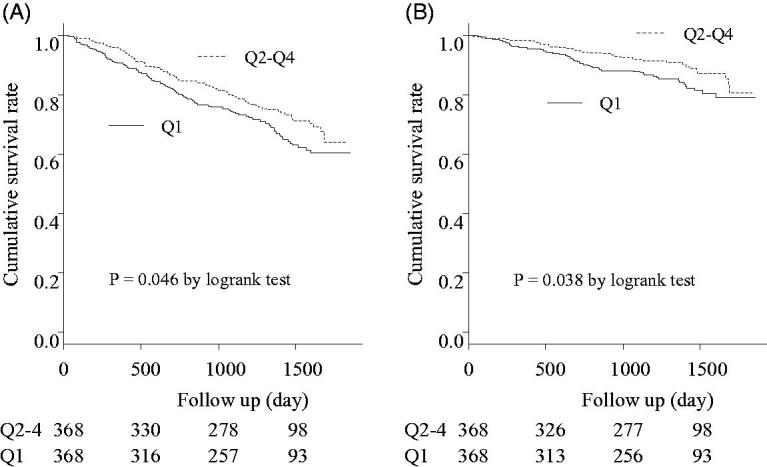
Kaplan-Meier curves for the cumulative survival rates between the Q1 group and the Q2–4 group after propensity-score matching. A: all-cause mortality Significant differences were observed between the two groups’ cumulative survival rates (*p* = .046). B: Cardiovascular related mortality Significant differences were observed between the two groups’ cumulative survival rates (*p* = .038).

There were 217 deaths during the follow-up period (Q1 group, 120 deaths; Q2–4 group, 97 deaths). Among them, there were 91 cardiovascular-related deaths during the follow-up period (Q1 group, 54 deaths; Q2–4 group, 37 deaths). Figure 3 displays the Kaplan-Meier curves for all-cause mortality in the two groups after propensity score-matching. All-cause and cardiovascular-related mortality were significantly higher in the Q1 group (*p* = .046 and *p* = .038, respectively). No significant difference for all-cause mortality was observed among the PS Q4 cohort (*p* = .342) (Supplementary Figure).

### 3.5. Hazard ratios for all-cause mortality among the propensity score-matched cohorts

Table 3 shows the results of multivariate analyses. All-cause mortality was significantly higher in the Q1 group than in the Q2–4 group after adjustment for age, gender, and history of CAD (hazard ratio [HR] = 1.31, 95% confidence interval [CI]: 1.00–1.72, *p* = .048]. However, there were no significant differences between the two groups after adjustment for eGFR (HR =1.21, 95% CI: 0.92–1.60, *p* = .181). Meanwhile, serum phosphate level as a continuous value was not significantly associated with all-cause mortality in any models.

**Table 3.** Associations of serum phosphate with all-cause mortality according to the multivariate cox proportional hazard regression analysis among the propensity score-matched cohorts.

		HR	95%CI	*p* value
Q1 (Q2–4 as reference)	Unadjusted	1.31	1.01–1.72	.046
	Model 1	1.32	1.01–1.72	.044
	Model 2	1.31	1.00–1.72	.048
	Model 3	1.21	0.91–1.59	.189
	Model 4	1.21	0.92–1.60	.181
Serum phosphate (per 1 mg/dL)	Unadjusted	0.89	0.79–1.00	.052
	Model 1	0.90	0.80–1.01	.084
	Model 2	0.90	0.80–1.02	.090
	Model 3	0.96	0.85–1.08	.487
	Model 4	0.96	0.84–1.08	.465

Model 1: adjusted for age and gender; Model 2: Model 1 plus adjusted for history of CAD; Model 3: Model 1 plus adjusted for eGFR; Model 4: Model 1 plus history of CAD and eGFR.

### 3.6. Stratified analysis with and without the use of calcium carbonate

**Figure 4.**
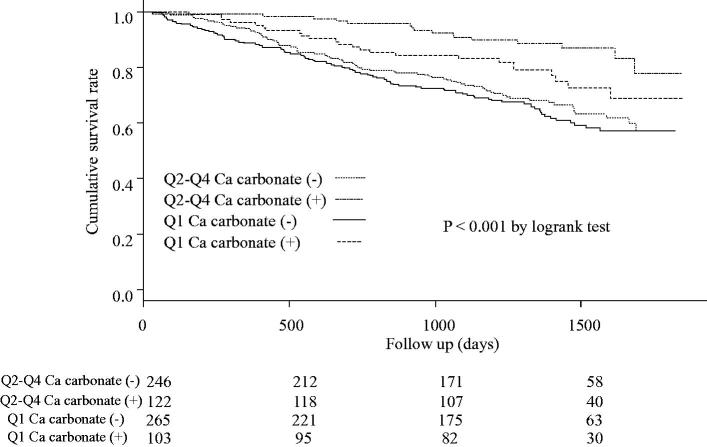
Kaplan-Meier curves for the cumulative survival rates between the Q1 group and the Q2–4 group after propensity-score matching stratified by use or no use of calcium carbonate. Significant differences were observed between the four groups’ cumulative survival rates (*p* < .001).

Figure 4 compares all-cause mortality among stratified groups with and without the use of calcium carbonate as a phosphate binder. Significant differences were observed in the cumulative survival rates between the four groups (*p* < .001).

## 4. Discussion

The present results revealed serum phosphate levels at dialysis initiation to be associated with all-cause and cardiovascular-related mortality after dialysis initiation. We would like to highlight the following features of this study: to our knowledge, this is the first study to examine the relationship between serum phosphate level at the time of dialysis initiation and all-cause mortality among dialysis patients after propensity score-matching. However, we could not verify that the serum phosphate level at dialysis initiation as an independent risk factor for all-cause mortality during maintenance dialysis because serum phosphate level was dependent of renal function.

Previous reports indicated a J-shaped relationship between serum phosphate level and mortality in patients on maintenance dialysis [5–7,14–17]. Selamet et al. indicated that serum phosphate level was associated with the mortality and cardiovascular events in patients with CKD stages 3–5 [18]. Higher serum phosphate levels cause vascular calcification, while lower levels represent a poor nutritional state. We matched the Q1 group and the Q2–Q4 group by propensity score because the Q1 group presented the highest all-cause mortality. Block et al. demonstrated that groups with lower or higher serum phosphate levels had significantly higher mortality than those with serum phosphate level of 4.0–5.0 mg/dL [5]. In a Japanese cohort, Taniguchi et al. showed that groups with serum phosphate levels of 5.0–6.0 mg/dL or more and less than 3.5–4.0 mg/dL had significantly higher mortality in some models [6]. We showed that the Q1 group, which presented the lowest serum phosphate level, had higher all-cause mortality than the other groups. However, the serum phosphate level, which was 4.3 mg/dL on average, was not as high in the Q1 group after propensity score-matching. Significant differences between the two groups after matching were not observed in the serum albumin level and BMI. Hence, we considered the low serum phosphate level to indicate malnutrition. There were significant differences in history of CAD and renal function including eGFR and serum creatinine level, even after propensity score-matching. Serum phosphate concentration increased as renal function decreased, especially in patients with GFR of less than 30 mL/min/1.73m^2^ [18]. We considered the timing of dialysis initiation to be strongly associated with serum phosphate level at dialysis initiation. In other words, patients with a history or comorbidity of cardiovascular disease including CAD were likely to begin dialysis earlier. Therefore, the serum phosphate level in those patients had not yet increased significantly. We could not find the association with mortality analysis with some variables including serum phosphate level as a continuous variable. We considered the reasons as the following. According to the increase in serum phosphate level, mortality did not decrease, but prognosis was poorer in only patients with lower phosphate level. Moreover, the average age of the Q1 group was much higher than those of the other groups. We showed that all-cause mortality was significantly higher in the Q1 group among patients with the use of calcium carbonate as a phosphate binder. Lower serum phosphate level in patients with the use of calcium carbonate was also associated with higher eGFR at dialysis initiation. We surmised that serum phosphate level was dependent on renal function rather than the use of calcium carbonate at dialysis initiation. Generally, the use of calcium-containing phosphate binders is not recommended because of the possibility of causing vascular calcification [19–24]. This study used calcium carbonate, which was only available in Japan during the registration period of this study. It was possible that the use of calcium carbonate to decrease serum phosphate levels at dialysis initiation was associated with lower all-cause mortality. However, use of non-calcium containing phosphate binders may be associated with lower all-cause mortality than calcium-containing phosphate binders.

The present study had the following limitations. First, although we adjusted for confounding factors during the analyses, the observational study design led to the differences in the patient characteristics. Residual confounding is a possibility, despite the use of propensity score-matched cohorts. Second, the timing of dialysis initiation varied among the patients as it was solely based on the attending physicians' judgment. The timing for initiating dialysis changes over time, depending on the available evidence, which is based on various guidelines of clinical management. Nevertheless, given that nephrologists decided on the timing for initiating dialysis in all the subjects in this study and the decisions on when to initiate dialysis were made in the same historical period, major differences between institutions and attending physicians were unlikely. Third, the use of phosphate binders and the duration of therapy before dialysis initiation were unclear.

We conclude that the serum phosphate level at the time of dialysis initiation was associated with all-cause mortality. However, the serum phosphate level was dependent on renal function.
